# Development and evaluation of time‐resolved fluorescent immunochromatographic assay for quantitative detection of SARS‐CoV‐2 spike antigen

**DOI:** 10.1002/jcla.24513

**Published:** 2022-06-12

**Authors:** Buzhou Xu, Hao Tang, Yiming Weng, Valerie Sloane Jones, Shuhong Luo, Chih Yun Cho, Yongping Lin, Jianmin Fang, Xuedong Song, Ruo‐Pan Huang

**Affiliations:** ^1^ RayBiotech, Guangzhou Guangzhou China; ^2^ RayBiotech Life Peachtree Corners Georgia USA; ^3^ The First Affiliated Hospital of Guangzhou Medical University Guangzhou China; ^4^ South China Biochip Research Center Guangzhou China

**Keywords:** COVID‐19, RT‐PCR, SARS‐CoV‐2, Spike protein, time‐resolved fluorescence, TRF lateral flow

## Abstract

**Background:**

The spread of COVID‐19 worldwide caused by the severe acute respiratory syndrome coronavirus 2 (SARS‐CoV‐2) has necessitated efficient, sensitive diagnostic methods to identify infected people. We report on the development of a rapid 15‐minute time‐resolved fluorescent (TRF) lateral flow immunochromatographic assay for the quantitative detection of the SARS‐CoV‐2 spike protein receptor‐binding domain (S1‐RBD).

**Objectives:**

Our objective was to develop an efficient method of detecting SARS‐CoV‐2 within 15 min of sample collection.

**Methods:**

We constructed and evaluated a portable, disposable lateral flow device, which detected the S1‐RBD protein directly in nasopharyngeal swab samples. The device emits a fluorescent signal in the presence of S1‐RBD, which can be captured by an automated TRF instrument.

**Results:**

The TRF lateral flow assay signal was linear from 0 to 20 ng/ml and demonstrated high accuracy and reproducibility. When evaluated with clinical nasopharyngeal swabs, the assay was performed at >80% sensitivity, >84% specificity, and > 82% accuracy for detection of the S1‐RBD antigen.

**Conclusion:**

The new S1‐RBD antigen test is a rapid (15 min), sensitive, and specific assay that requires minimal sample preparation. Critically, the assay correlated closely with PCR‐based methodology in nasopharyngeal swab samples, showing that the detected S1‐RBD antigen levels correlate with SARS‐CoV‐2 virus load. Therefore, the new TRF lateral flow test for S1‐RBD has potential application in point‐of‐care settings.

## Graphical abstract

We have developed a portable, disposable, and quantitative lateral flow device, which detects the SARS‐CoV‐2 Spike antigen in 15 min using time‐resolved fluorescence. Compared with RT‐PCR, this assay has a sensitivity of 80.3% and a specificity of 83.64% in a cohort of 293 patients.

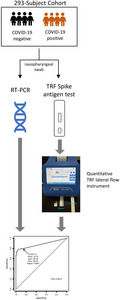

## 1 INTRODUCTION

The COVID‐19 pandemic, caused by severe acute respiratory syndrome coronavirus 2 (SARS‐CoV‐2), has sustained its catastrophic effects worldwide. The most effective measures to mitigate the spread of COVID‐19 are efficient screening, detection, and isolation of the infected persons. This detection/isolation approach necessitates simple, rapid, portable, and low‐cost detection tools, so that infected persons can be identified and isolated immediately.

Laboratory testing methods for SARS‐CoV‐2 have been extensively reviewed.^1^, ^2^, ^3^, ^4^, ^5^, ^6^, ^7^, ^8^, ^9^, ^10^, ^11^, ^12^, ^13^, ^14^, ^15^, ^16^, ^17^ Currently, there are three major detection methods that have been commercialized under Emergency Use Authorization (EUA) from the U.S. Food and Drug Administration (FDA), each with its own merits and limitations. The most widely used diagnostic detection methods are based on detection of viral nucleic acid^1^, ^4^, ^8^, ^9^, ^10^, ^12^, ^13^, ^15^, ^16^, ^17^, ^18^ such as real‐time reverse transcription‐polymerase chain reaction (RT‐PCR) and loop‐mediated isothermal amplification. While these methods are highly sensitive and specific, the long processing time (3–4 h) coupled with the need for costly, specialized instrumentation, and personnel training have imposed considerable challenges on high‐volume COVID‐19 testing laboratories, resulting in highly variable turnaround times of 2–7 days. As a result, infected persons may continue to engage in social activities and potentially transmit SARS‐CoV‐2 to others in the period between sampling and report of detection results. Furthermore, nucleic acid tests may report a positive result, even when the virus is not viable and the patient is no longer contagious.

Serological assays detect antibodies produced from the immune response to SARS‐CoV‐2 rather than its genetic signature and have been widely used in COVID‐19 diagnostics.^9^, ^18^, ^19^, ^20^, ^21^ Immunochromatographic (lateral flow) tests have been particularly favored for their low cost, rapidity, ease of use,^22^, ^23^, ^24^ and applicability to point of care (POC). Lateral flow antibody tests have proven useful for screening, tracking, and monitoring individuals who have been exposed to SARS‐CoV‐2. However, IgM or IgG antibodies are not detectable in the infected person until many days or even weeks after infection. IgM is typically detectable in an infected person earlier (2–5 days post‐exposure) and gradually decrease while IgG levels increase. Thus, serological tests often fail to detect an early‐phase infection.

The third type of diagnostic immunoassay is the antigen test, which relies upon detection of one or more SARS‐CoV‐2 signature proteins.^25^, ^26^, ^27^, ^28^ The spike protein (S‐protein) and nucleocapsid are the typical targets of COVID‐19 antigen tests. Several methods have been developed to detect and quantify SARS‐CoV‐2 antigens, including chemiluminescence‐based immunoassay,^29^, ^30^ electro‐chemiluminescence‐based immunoassay, and lateral flow immunoassay.^31^, ^32^, ^33^ Like lateral flow antibody tests, lateral flow antigen tests have enjoyed considerable popularity for their ease of use, low cost, and applicability to POC and settings with limited resources. However, they have limited sensitivity and are best applied only for patient screening rather than for final diagnosis. In addition to the three test methods described above, several new detection techniques have been reported. Biosensor‐based detection methods are of particular interest because they have potential to provide rapid and sensitive detection, in some cases, without destroying samples.^34^, ^35^, ^36^, ^37^ However, these techniques are currently not mature enough to implement in fighting the SARS‐CoV‐2 pandemic.

While less sensitive than PCR, antigen tests have emerged as the method of choice for rapid SARS‐CoV‐2 screening, as well as for point‐of‐care and home testing. Several commercial rapid antigen tests have been recently evaluated for their detection limits in different matrices, revealing that some tests failed to correctly diagnose patients with low viral loads.^38^ To overcome this limitation, novel detection techniques have been explored more recently. Lee. et al. developed a novel rapid detection method for SARS‐CoV‐2 spike 1 protein using the SARS‐CoV‐2 receptor ACE2 along with commercially available antibodies, which together formed a matched pair with good detection sensitivity.^39^ Molecularly imprinted polymers (MIPs) have also shown promise as a replacement for antibodies in quantitative detection of SARS‐CoV‐2 spike 1 protein.^40^ However, MIP generally lacks ideal binding affinity and specificity with the antigen, resulting in relatively low detection sensitivity. Surface‐enhanced Raman scattering (SERS)‐based probes have been investigated for both detection and registration of SARS‐CoV‐2 antigen. SERS‐based immunoassays generally do not exhibit matrix effects, enabling direct measurement of antigens without pre‐treatment of biological samples,^41^, ^42^ in contrast to conventional fluorescence detection methods.^43^, ^44^ Karakus et al.^45^ reported colorimetric and electrochemical detection of SARS‐CoV‐2 spike protein with gold nanoparticle‐based biosensors to detect as little as 1 pg/ml of the spike protein with linearity over four orders of magnitude. A unique method was recently reported that uses a bioelectric recognition assay‐based biosensor to detect spike protein on the viral surface. The assay is performed in only three minutes with high sensitivity and selectivity and was able to detect the virus in positive samples with a 92.8% success rate compared with RT‐PCR. This method may thus prove valuable in the fight against COVID‐19.^46^ Finally, Signal et al. demonstrated that rapid immunoassays can equally be useful for quantifying SARS‐CoV‐2 nucleocapsid (N) and spike (S) antigens in blood of pediatric patients with COVID‐19.^47^

Fluorescence‐based detection is commonly used for both PCR and immunoassays. Conventional fluorometry detects wavelength differences between fluorescence signals and background noise (e.g., scattered light of excitation photons, autofluorescence of samples matrices). Time‐resolved fluorescence (TRF), an alternative spectroscopic technique, excludes background noise by allowing signal decay following a brief pulse of excitation. The remaining long‐lived signal from the fluorescent label is then measured. The time delay between excitation and measurement results in lower background, higher signal/noise ratio, and potentially, higher detection sensitivity when long lifetime fluorescent probes are used.^48^, ^49^ It is well established that TRF detection technique can achieve higher signal–noise ratio than conventional fluorescence techniques (up to two orders of magnitude). Additionally, TRF does not require expensive band‐pass optical filters for signal–noise separation. It is thus feasible to construct portable, less expensive readers to detect TRF signals of long lifetime fluorescent probes.

For any immunoassay method, the antibody's binding affinity and specificity for the antigen are critical for overall test performance. Herein, we report the development of a rapid lateral flow‐based SARS‐CoV‐2 antigen test using antibodies against SARS‐CoV‐2 S‐protein receptor‐binding domain (S1‐RBD). Our methodology provides rapid, quantitative detection of S1‐RBD antigen in clinical nasopharyngeal swab samples and has potential utility for improved COVID‐19 screening in clinical settings.

## 2 MATERIALS AND METHODS

### 2.1 Materials

Mouse anti‐SARS‐CoV‐2 S1‐RBD antibodies (cat# 130‐108015 and cat# 130‐10814) and recombinant SARS‐CoV‐2 S1‐RBD antigen (cat# 230‐30162) were purchased from RayBiotech, Inc. Goat anti‐rabbit IgG (cat# P200301) and rabbit IgG (N160701) were purchased from Boyin Biotech Ltd, Hanzhou, China. Nitrocellulose membrane (cat# VIV12025100R) was from Life Sciences Pall Vivid 170. Latex particles (Cat# FCEU003) were from Bangs Laboratories. Disposable virus sampling tubes were from Dakewe BioSci.

### 2.2 Preparation of diluent buffer

About 45.96 g of disodium hydrogen phosphate, 11.84 g of sodium dihydrogen phosphate dihydrate, 36 g of sodium chloride, 8 g of casein, and 4 ml of ProClin™ 300 were dissolved in Milli‐Q water to a total volume of 400 ml with a final pH of 7.4

### 2.3 SARS‐CoV‐2 S1‐RBD antigen lateral flow assay design

The S1‐RBD assay requires a TRF reader. The instrument used herein was a single channel reader (Guangzhou Labsim Biotech Co., Ltd, model # AFS‐1000) whose detection principle has been described previously.^48^, ^49^

**FIGURE 1:**
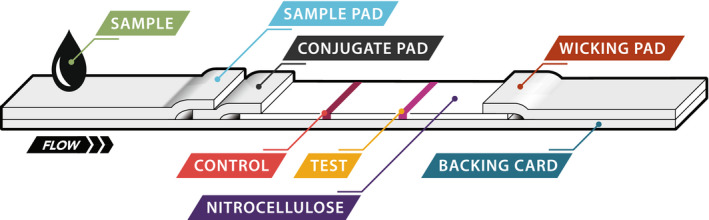
Schematic representation of antigen test strip

The procedures for constructing the SARS‐CoV‐2 S1‐RBD lateral flow device were similar to previously reported methods^50^ and consisted of three phases. First, antibody‐particle conjugates were prepared by activating latex particles containing surface carboxylic acid groups with 1‐ethyl‐3‐(3‐dimethylaminopropyl) carbodiimide, followed by covalent attachment of the antibodies to the particle surface. The conjugates were then washed and stored. Next, the individual components were prepared, including treating a sample pad with blocking agents and immobilizing detection antibodies and calibration antibodies at discrete locations on nitrocellulose membranes (the detection zone and calibration zone, respectively). Finally, the sample pad, nitrocellulose membrane, and wicking pad (Figure 1) were assembled into a plastic casing to create a disposable, single‐use test cassette.

### 2.4 Preparation of standard SARS‐CoV‐2 S1‐RBD antigen

Recombinant SARS‐CoV‐2 S1‐RBD antigen samples of various concentrations were prepared by diluting an appropriate amount of the antigen with diluent buffer into 100 μl in disposable virus sampling tubes. These standard antigen samples were measured in triplicate by the SARS‐CoV‐2 S1‐RBD assay.

### 2.5 Procedures for SARS‐CoV‐2 S‐RBD antigen measurements

The TRF reader was turned on and stabilized for 5 min before use. An ID card associated with each lot of cassettes was inserted and activated. After diluting an appropriate amount of sample with diluent buffer, 100 μl of diluted sample was applied to the sample zone of a test cassette. The test cassette was then immediately inserted into the cassette holder of the AFS‐1000 reader. The signal was collected and recorded 15 min after sample application.

### 2.6 Linearity and dynamic range study

A series of SARS‐CoV‐2 S1‐RBD antigen samples were prepared at the following concentrations: 0, 10, 30, 100, 300, 1000, 3000, 10,000, and 20,000 pg/mL. SARS‐CoV‐2 S1‐RBD antigen test cassettes from two lots were tested. For samples of each concentration, five test cassettes from each of the two lots were used for measurements by the AFS‐1000 reader. The signal for each test cassettes was collected three times.

### 2.7 Clinical samples

De‐identified patient nasopharyngeal swabs were purchased from PanoHealth, LLC. The specimens (swabs in viral transport medium [VTM] buffer) were thawed from −80°C immediately before use. PanoHealth LLC is certified under the Clinical Laboratory Improvement Amendments of 1988 (CLIA).

### 2.8 SARS‐CoV‐2 S1‐RBD assay and RT‐PCR

The test cassettes and sample diluents were warmed to room temperature before use. The VTM sample was mixed thoroughly with diluent and 100 μl of diluted VTM was added to the sample pad of the cassette. After developing for 15 min, the cassettes were immediately loaded into the TRF reader according to manufacturer's protocol. The testing of viral RNA in clinical swab samples was performed using a commercial SARS‐CoV‐2 RT‐PCR kit according to the manufacturer's protocol (Thermo Fisher, cat# A47814).

## 3 RESULTS

### 3.1 Development of SARS‐CoV‐2 S1‐RBD antigen test

The SARS‐CoV‐2 antigen test described here has two components including the consumable test cassettes (S1‐RBD cassette) and the TRF reader. The test is based on the immunochromatographic sandwich assay principle. The assembly of the test cassettes has been described in detail.^50^ A schematic depiction of the S1‐RBD antigen test strip housed in a plastic cassette is shown in Figure 1. Its configuration is slightly different from commercial or previously reported lateral flow immunochromatographic devices in that the calibration zone is placed upstream of sample flow and the detection zone is placed downstream of sample flow. Our study has found that the upstream positioning of the control zone provides more consistent and reliable calibration than when the zone is placed downstream. The S1‐RBD cassette uses europium particle‐based conjugates that provide a long lifetime fluorescence signal (>500 μs). The maximal fluorescence peak of the europium particle is 615 nm, and the maximal absorption peak is around 375 nm with a Stoke shift of 240 nm. Two different particle conjugates are used for generation of the calibration and detection signals and are co‐deposited in the conjugate pad.

Several monoclonal antibodies were screened for utility as particle conjugates and as capture reagents on nitrocellulose membranes. Of all antibodies tested, mouse anti‐SARS‐CoV‐2 S1‐RBD monoclonal antibody (RayBiotech, cat# 130‐10814) performed best when covalently attached to the europium latex particles to make detection conjugates. Mouse anti‐SARS‐CoV‐2 S1‐RBD monoclonal antibody (RayBiotech, cat# 130‐10815) performed with high affinity as capture agents. Goat anti‐IgG covalently attached on the surface of europium latex particles bound with high affinity to anti‐S1‐RBD monoclonal antibody. The conjugates were used for generation of the calibration signal while rabbit IgG was immobilized to form a calibration zone.

The detection principle of the AFS‐1000 TRF reader was reported previously.^48^, ^49^ Briefly, the TRF detection technique provides high signal–noise ratio with a potential for high detection sensitivity due to the long fluorescence lifetime and large Stoke shift of the europium particles. The AFS‐1000 instrument uses an LED of 375 nm as the excitation light source and silicon diodes for fluorescence measurement. Rather than costly band‐pass optical filters, the TRF reader uses an inexpensive cutoff filter and gated timing circuit to measure fluorescence signals. Results are generated 15 min after sample application to the S1‐RBD cassette.

### 3.2 Linearity and dynamic range

**FIGURE 2:**
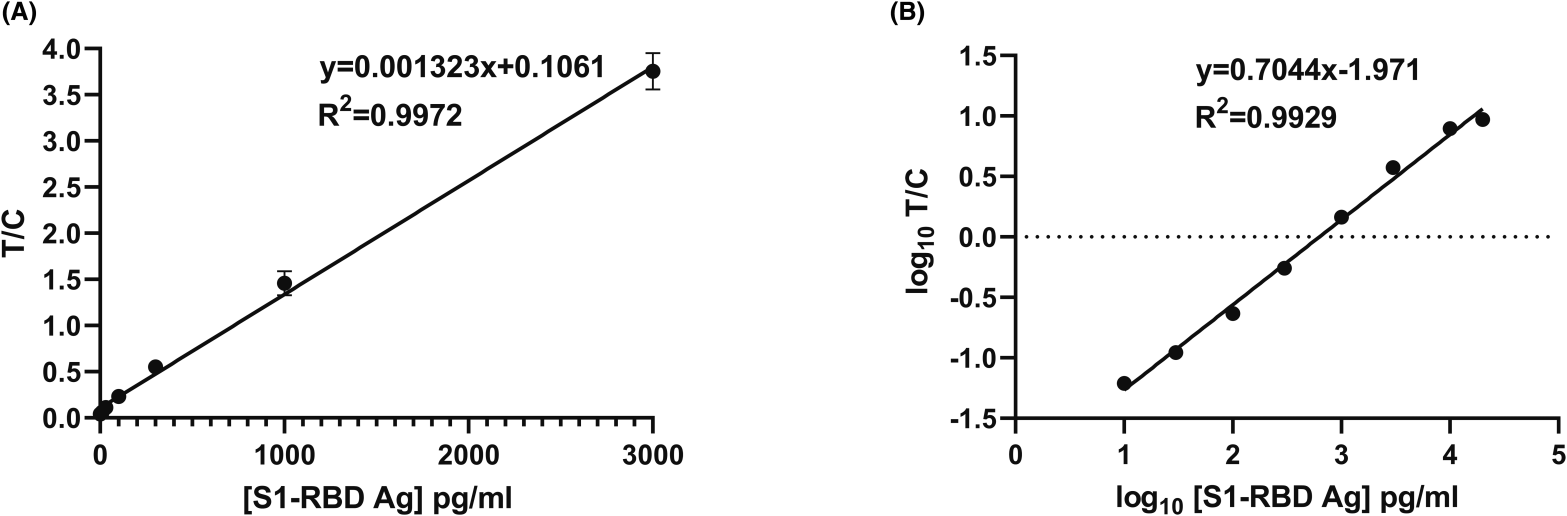
T/C signals as a function of S1‐RBD Ag concentration up to 3000 pg/ml (A). Log_10_ (T/C) signals as a function of log_10_ (S1‐RBD antigen concentration) up to 20,000 pg/ml (B). Error bars represent standard deviation (SD) for quadruplicate readings

To evaluate batch‐to‐batch reproducibility, two independently generated lots (lot 1 and 2) of S1‐RBD cassettes were investigated. TRF signals from the cassettes are expressed as the ratio (T/C) of signal in the detection zone (T) to signal in the calibration zone (C). For both lots, T/C signal had a linear relationship up to 3000 pg/ml S1‐RBD (Figure 2). The performance of the two lots was similar. T/C values for a representative lot are shown in Table 1. The T/C signal is linear up to 3000 pg/ml (Figure 2A), but the linearity range was extended up to 20,000 pg/ml with log transformation of T/C (Figure 2B).

**TABLE 1:** T/C signals measured by 4 different cassettes (lot 1) for S1‐RBD antigen of various concentrations

S1‐RBD (pg/ml)	0	10	30	100	300	1000	3000	10,000	20,000
T/C	0.0469	0.0629	0.1136	0.2159	0.5945	1.626	3.940	7.623	9.266
	0.0408	0.0663	0.1022	0.2480	0.5570	1.449	3.901	8.008	9.040
	0.0433	0.0606	0.1036	0.2307	0.5667	1.303	3.541	8.296	9.603
	0.0389	0.0565	0.1233	0.2353	0.4895	1.459	3.638	7.524	9.529
AVG	0.0425	0.0616	0.1107	0.2325	0.5519	1.459	3.7550	7.863	9.360
SD	0.0035	0.0041	0.0098	0.0133	0.0445	0.1321	0.1958	0.3564	0.2575
CV	8.14%	6.68%	8.88%	5.70%	8.07%	9.05%	5.21%	4.53%	2.75%

### 3.3 Assay accuracy

**FIGURE 3:**
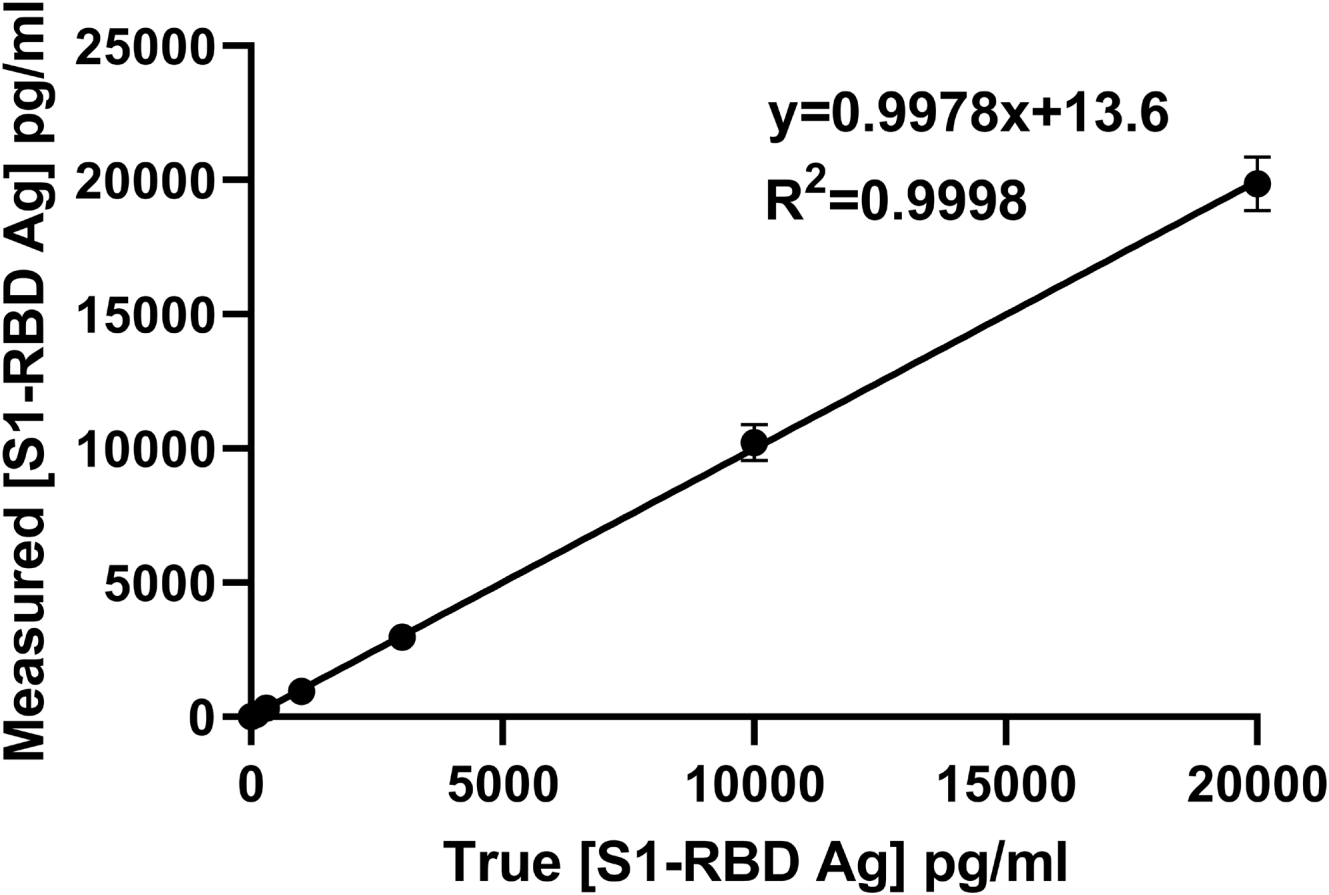
Measured (observed) S1‐RBD Ag concentrations versus the true concentration (4 cassettes of lot 1). Error bars represent standard deviation (SD) for four replicates

Correlation between measured and true concentrations of a series of S1‐RBD antigen standards was studied for the S1‐RBD cassettes from lot 1 and lot 2. Measured concentrations for a series of standards are shown in Table 2. Each concentration was determined by the TRF reader for four cassettes of lot 1 and 2. The data closely fitted the regression line as exhibited by an *R*‐squared value of .9998 and .9967 for lot 1 and lot 2, respectively (Figure 3).

**TABLE 2:** Measured concentrations for a series of S1‐RBD Ag standards determined by 4 different cassettes (lot 1)

S1‐RBD (pg/ml)	10	30	100	300	1000	3000	10,000	20,000
Measured concentration	11.50	28.00	102.06	336.05	956.54	2747.06	10,875.30	19,787.88
	10.21	28.13	107.96	293.87	1072.82	2970.83	9676.95	20,941.44
	9.19	28.99	91.42	324.63	862.88	3147.41	9627.37	18,543.03
	9.59	31.94	111.56	305.18	909.83	3047.83	10,730.82	20,162.93
AVG	10.1	29.27	103.25	314.93	950.52	2978.28	10,227.61	19,858.82
SD	1.01	1.837	8.805	18.963	90.055	170.255	667.392	1000.147
CV	10.0%	6.28%	8.53%	6.02%	9.47%	5.72%	6.53%	5.04%

To further investigate the accuracy of the S1‐RBD antigen test, three recombinant S1‐RBD solutions with concentrations of 100, 300, and 1000 pg/mL were tested in triplicate on two lots of S1‐RBD cassettes. Both lots showed less than 9% deviation from true antigen concentration (Table 3).

**TABLE 3:** Comparison of average measured (observed) S1‐RBD concentrations (lot 1) with true S1‐RBD concentrations

True [S1‐RBD] (pg/ml)	100	300	1000
Avg. measured [S1‐RBD] (pg/ml)	96.0	280	926
CV	4.21%	3.82%	8.37%
Accuracy	−4.01%	−6.53%	−7.31%

### 3.4 Assay sensitivity and precision

A series of diluted recombinant S1‐RBD antigen solutions were measured using two lots of S1‐RBD cassettes (20 cassettes per lot). Detection limit was defined as the antigen concentration corresponding to the average experimental value (T/C signal) at 0 pg/ml plus 2× standard deviation (SD). Based on the calibration curve of the two lots, the detection limits were 5.2 pg/ml (lot 1) and 6.6 pg/ml (lot 2), respectively.

For precision analysis, three recombinant S1‐RBD solutions at concentrations of 100, 300, and 1000 pg/ml were used. Two lots of S1‐RBD cassettes were studied using 10 tests from each lot. Their coefficients of variation (CV) were found to be 8.7% and 11%, respectively, for 100 pg/ml; 12%, and 7.3%, respectively, for 300 pg/ml; and 4.2% and 7.3%, respectively, for 1000 pg/ml. The two lots demonstrated an SD of <13%.

### 3.5 Assay hook effect

**FIGURE 4:**
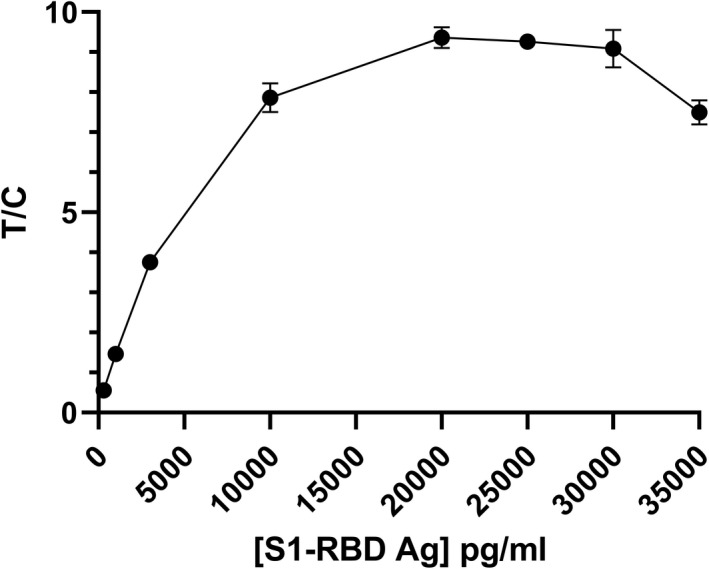
Plot of TRF signals vs SARS‐CoV‐2 S1‐RBD concentrations. Error bars represent standard error of the mean (SEM) for quadruplicate readings

The S1‐RBD antigen test uses sandwich immunoassay methodology which can potentially exhibit the characteristic “hook effect” at high antigen concentrations. To evaluate the hook effect of the assay, a dilution series of recombinant S1‐RBD was prepared: 300 pg/ml, 1 ng/ml, 3 ng/ml, 10 g/ml, 20 ng/ml, 25 ng/ml, 30 ng/ml, and 35 ng/ml. Each solution was measured four times using four tests to obtain an average signal. Results revealed that the hook effect from these assays occurs at concentrations higher than 20 ng/ml (Figure 4).

### 3.6 Evaluation of diagnostic performance

The performance of the S1‐RBD antigen test was compared with a commercial SARS‐CoV‐2 RT‐PCR‐based testing kit (Thermo Fisher) using a cohort of 293 de‐identified clinical nasopharyngeal swabs. Inclusion criteria for COVID‐19‐positive swab samples were positive cases (confirmed by RT‐PCR) with COVID‐19 symptoms. The inclusion criteria for negative samples were cases with no symptoms and confirmed negative by RT‐PCR. Exclusion criteria were patients with unclear clinical diagnosis or with insufficient sample for testing. The 293 swabs were labeled randomly from sample 1 to sample 293. Of the 293 swabs, 183 were cases with positive diagnosis of COVID‐19 and the remaining 110 were cases with negative diagnosis. All swab samples were simultaneously tested by the SARS‐CoV‐2 S1‐RBD antigen test and by the commercially available RT‐PCR testing kit according to manufacturer's protocol.

**FIGURE 5:**
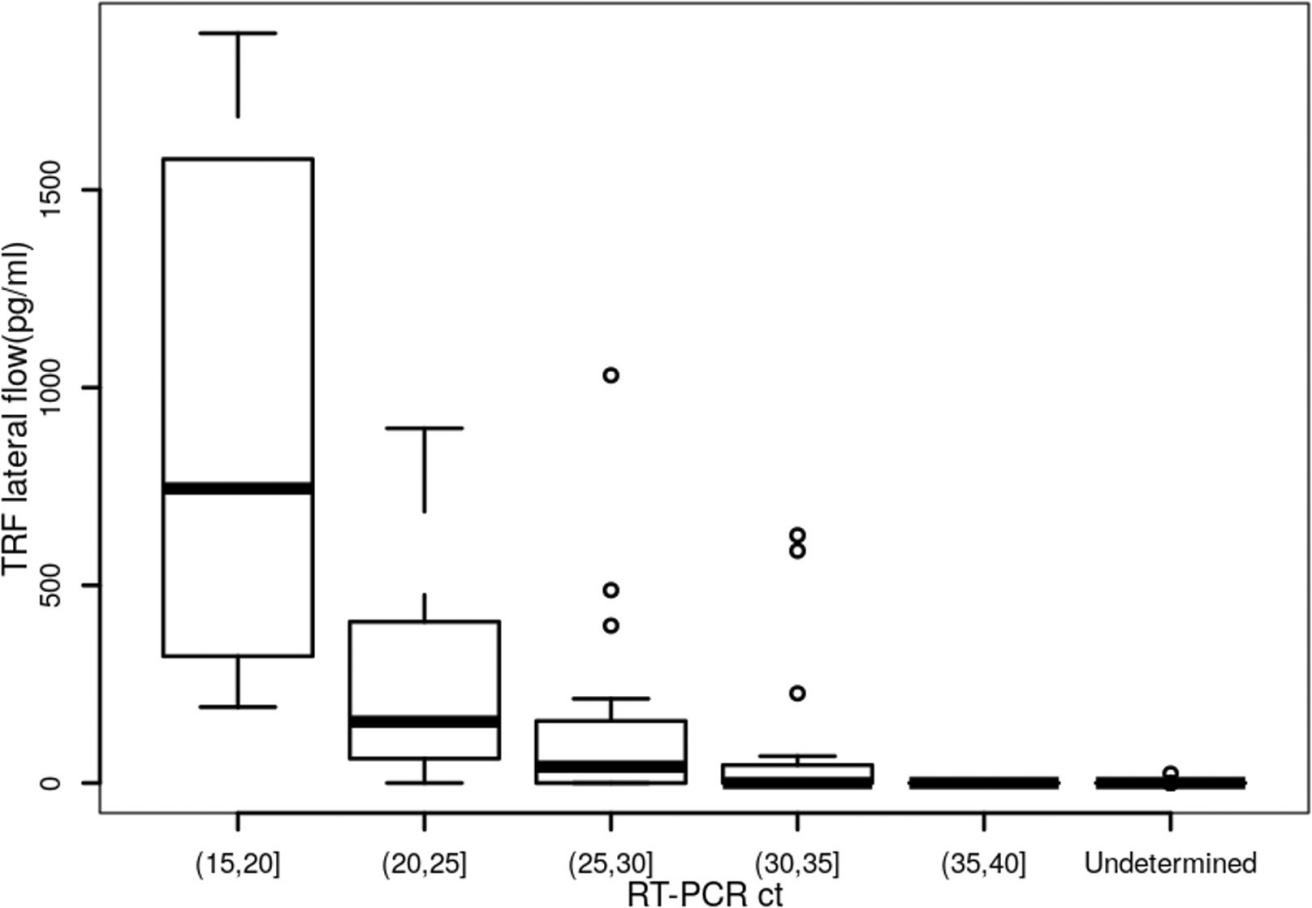
Correlation between SARS‐CoV‐2 RNA amount (Ct) detected by RT‐PCR (*x*‐axis) and SARS‐CoV‐2 S1‐RBD abundance detected by TRF lateral flow assay (*y*‐axis) in nasopharyngeal swab samples. The *ρ* value was −.76 and *p* value was <.001

We then compared the measured S1‐RBD antigen levels (pg/ml) generated from the S1‐RBD antigen test with the RT‐PCR Ct value collected from the same samples (Figure 5). Significant correlation between the S1‐RBD antigen test and RT‐PCR was detected with *ρ* value of −.76 and *p* value of <2.2e‐16 (<.001).

**FIGURE 6:**
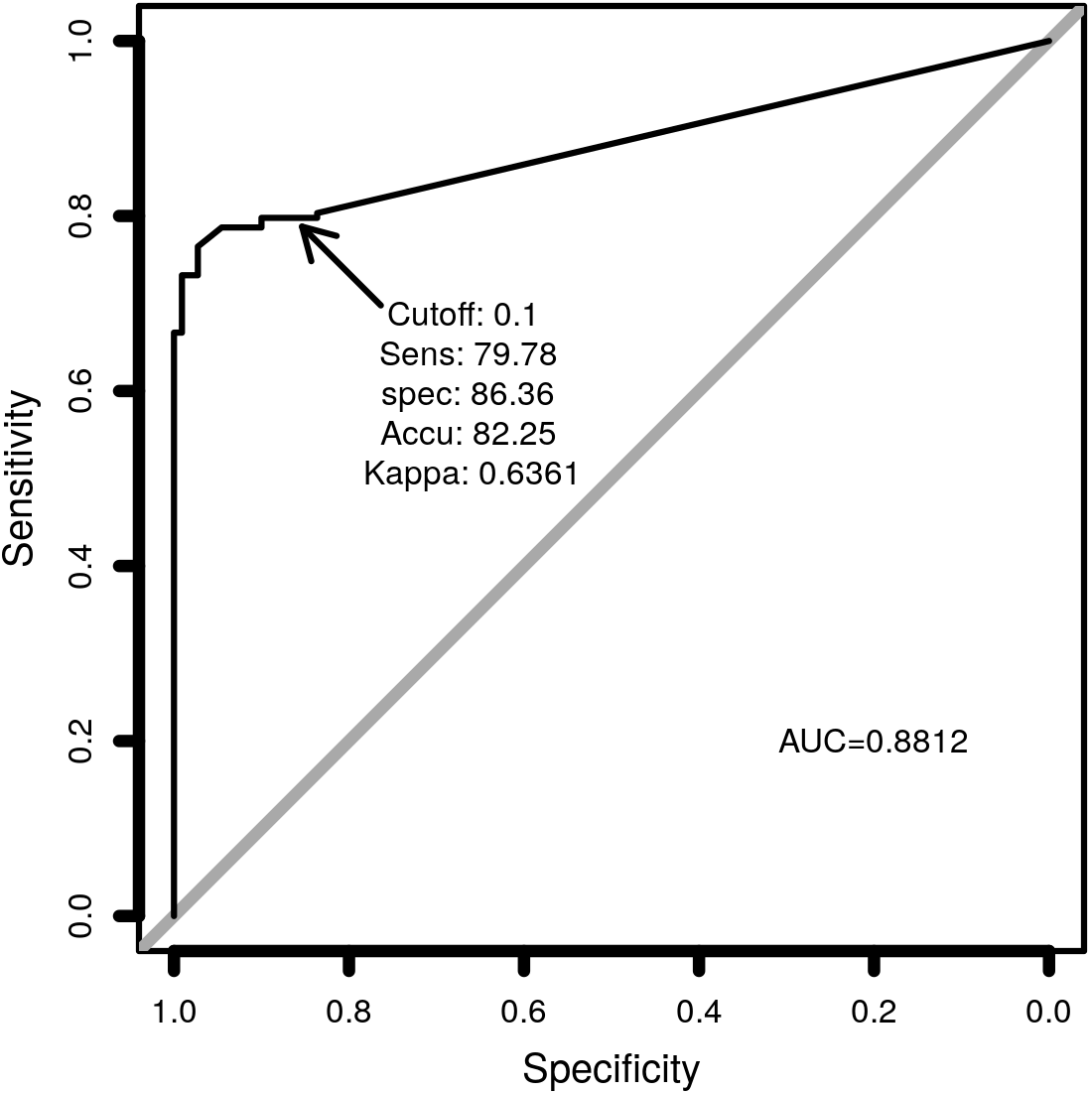
ROC curve of SARS‐CoV‐2 S1‐RBD antigen in nasopharyngeal swab samples

Finally, we undertook receiver operating characteristic (ROC) analysis comparing the measured S1‐RBD antigen levels (pg/ml) generated from the S1‐RBD antigen test with the RT‐PCR Ct value generated from the same samples (Figure 6). The sensitivity of the antigen test is 80.33% (147/183, 95% confidence interval: 73.82%–85.83%). The specificity is 83.64% (92/110, 95% confidence interval: 75.38%–90%). The accuracy is 81.57% (239/293, 95% confidence interval: 76.65%–85.84%) with a kappa value of .619. With an optimal cutoff point of 0.01 pg/ml, and the diagnostic results of the antigen test are compared with that of RT‐PCR for the 293‐sample cohort (Table 4).

**TABLE 4:** Diagnostic performance of SARS‐CoV‐2 S1‐RBD antigen test (TRF) vs. RT‐PCR in nasopharyngeal swabs

	Positive diagnosis (RT‐PCR)	Negative diagnosis (RT‐PCR)	Total
TRF+	147	18	165
TRF−	36	92	128
Total	183	110	293

Abbreviations: TRF+, Positive diagnosis of COVID‐19 by TRF antigen test; TRF−, negative diagnosis of COVID‐19 by TRF antigen test.

## 4 DISCUSSION

RT‐PCR analysis remains the gold standard for COVID‐19 diagnosis owing to its high sensitivity and ability to directly detect the virus, enabling detection in recently exposed individuals. However, the technique requires technical expertise, laborious RNA extraction from swab specimens, and a long workflow, which can contribute to significant resulting delays in high‐volume laboratories. These challenges have spurred interest in direct antigen detection due to the simplicity of immunoassays, particularly immunochromatographic techniques.

This report describes development and evaluation of a rapid SARS‐CoV‐2 antigen detection test for diagnosis of patients with COVID‐19. The methodology consists of a portable, disposable immunochromatographic test cassette, and an automated TRF analyzer with rapid reading capability. The assay was designed to target the S‐protein because of its high degree of conservation across SARS‐CoV‐2 variants^51^ and its uniqueness to the *Coronaviridae* family, which precludes cross‐reactivity with other viruses.

The assay is capable of rapidly quantifying both recombinant S1‐RBD and S‐protein antigen in clinical nasopharyngeal swab samples with a detection limit of less than 10 pg/ml. The TRF signal was linear from 0 to 20 ng/ml, and the assay demonstrated high accuracy and reproducibility at various concentrations. Most importantly, the assay correlated well with the gold standard RT‐PCR in clinical nasopharyngeal samples, suggesting that the S‐protein antigen signature correlates with high virus load.

The S1‐RBD antigen test assay has unique advantages. The protocol is simple, requiring only minimal training, using a portable, inexpensive cassette, and delivering results within 15 min. These features are critically important for point of care and settings with limited resources. Together, the TRF lateral flow‐based S1‐RBD antigen assay represents a convenient, rapid, inexpensive alternative to viral nucleic acid detection by RT‐PCR.

## CONFLICT OF INTEREST

We certify that some authors (Buzuo Xu, Hao Tang, Valerie Jones, Shuhong Luo, Chih Yun Cho, Jianmin Fang, and Ruo‐Pan Huang) are employees of and have a financial stake in RayBiotech. All other authors (Yiming Weng, Yongping Lin, and Xuedong Song) have no conflicts of interest.

## Data Availability

The data supporting these findings are available from the corresponding authors upon reasonable request.
